# Supplementary material for: Change in RhoGAP and RhoGEF availability drives transitions in cortical patterning and excitability in Drosophila
Source: bioRxiv. 2023 Nov 6:2023.11.06.565883. Preprint. [Version 1] doi: 10.1101/2023.11.06.565883 (PMC10659369; doi:10.1101/2023.11.06.565883)
Supplement: 1 [file NIHPP2023.11.06.565883V1-supplement-1.pdf]

bioRxiv preprint doi: https://doi.org/10.1101/2023.11.06.565883; this version posted November 6, 2023. The copyright holder for this preprint (which was not certified by peer review) is the author/funder, who has granted bioRxiv a license to display the preprint in perpetuity. It is made available under aCC-BY 4.0 International license.

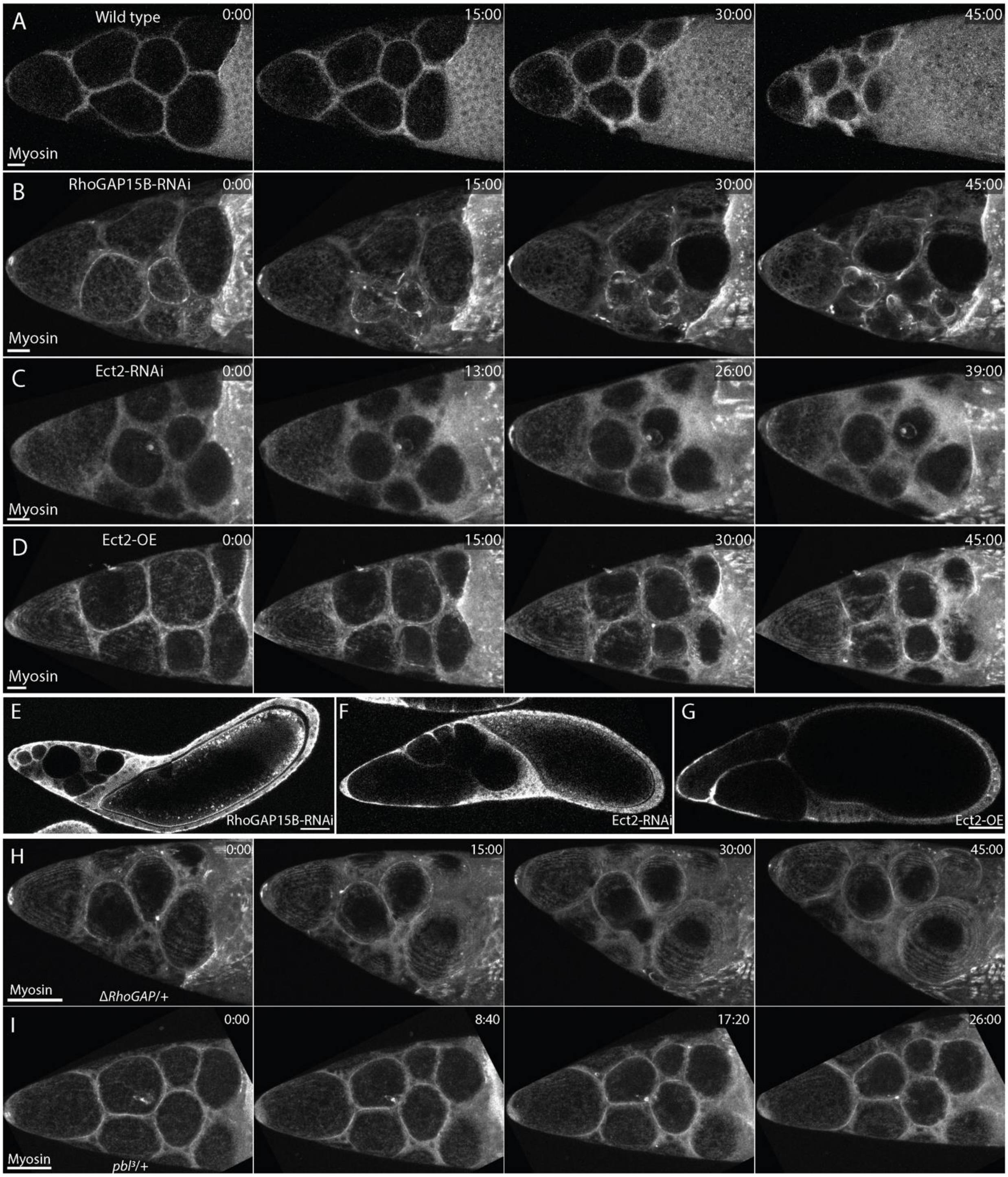

Supplemental Figure S1: Ect2 and RhoGAP15B perturbations block nurse cell dumping.

A. MIPs of myosin signal in a wild-type egg chamber showing reduction in cell volume and appearance of nonuniform myosin intensity in the third and fourth time points. **B**. MIPs from the same egg chamber as in Figure 1D, showing dumping stalling as erratic waves form in the smaller cells in the egg chamber. **C**. MIPs from the same egg chamber as in Figure 1E, showing dumping stalling without large-scale wave formation. **D**. MIPs from the same egg chamber as in

bioRxiv preprint doi: https://doi.org/10.1101/2023.11.06.565883; this version posted November 6, 2023. The copyright holder for this preprint (which was not certified by peer review) is the author/funder, who has granted bioRxiv a license to display the preprint in perpetuity. It is made available under aCC-BY 4.0 International license.

Figure 1F. Note the reduction in nurse cell cluster size in **A** that is not seen to the same degree in **B**-**D**. **E**-**G**. Images of egg chambers after dumping has stalled for (**E**) RhoGAP15B-RNAi, (**F**) Ect2-RNAi, and (**G**) Ect2-OE egg chambers. **E** and **F** are single optical sections from near the midplane, while **G** is a maximum-intensity projection. **H**, **I**. MIPs of myosin signal in egg chambers heterozygous for an allele of *RhoGAP15B* missing its RhoGAP domain (abbreviated as *ΔRhoGAP*) or for pbl^3, a null allele of Ect2. Scale bars: 20 µm in **A**-**D**; 50 µm in **E**-**I**. Time stamps are min:sec.

bioRxiv preprint doi: https://doi.org/10.1101/2023.11.06.565883; this version posted November 6, 2023. The copyright holder for this preprint (which was not certified by peer review) is the author/funder, who has granted bioRxiv a license to display the preprint in perpetuity. It is made available under aCC-BY 4.0 International license.

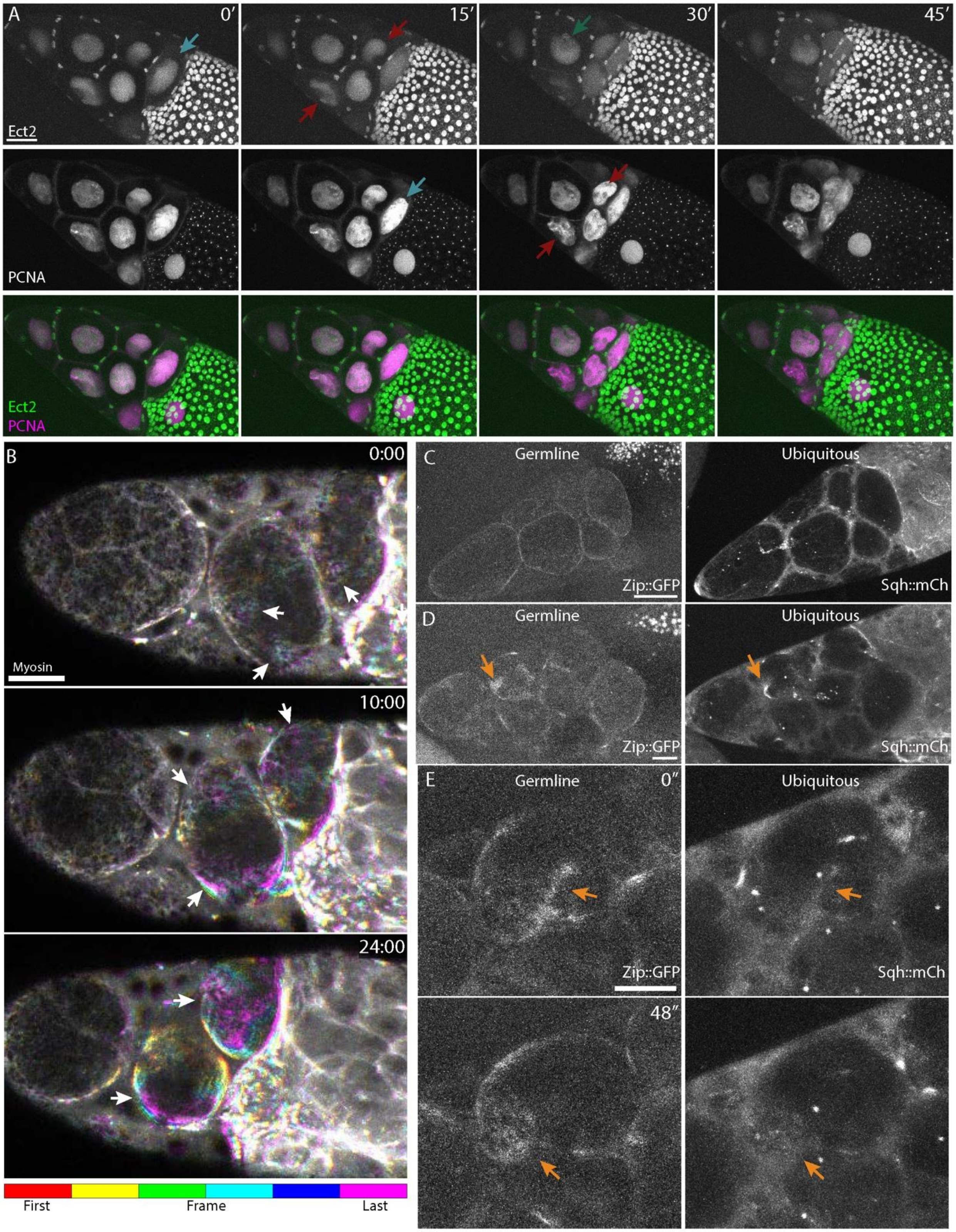

Supplemental Figure S2: PCNA serves as a proxy for Ect2 release from the nucleus as myosin gradually becomes more wave-like.

bioRxiv preprint doi: https://doi.org/10.1101/2023.11.06.565883; this version posted November 6, 2023. The copyright holder for this preprint (which was not certified by peer review) is the author/funder, who has granted bioRxiv a license to display the preprint in perpetuity. It is made available under aCC-BY 4.0 International license.

A. MIPs of Ect2 (top row) and PCNA (middle row) during dumping for one representative egg chamber, with merged image in the bottom row. Colored arrows point to approximate exit from the nucleus, with blue, red, or green arrows corresponding to cells one, two, or three ring canals from the oocyte, respectively. PCNA exits the nucleus roughly 10-15 minutes after but in the same order as Ect2. **B**. Three MIPs of myosin signal in an egg chamber with six subsequent frames, spanning one minute total, shown in different colors. From an initially uniform cortical distribution, myosin first shows temporary spatial accumulations or 'flickers' (top panel, arrows). Later in dumping, the flickers develop into faint waves (middle panel, arrows) that become progressively more persistent and intense (bottom panel, arrows). **C**. Germline-specific myosin signal (myosin heavy chain, Zipper::GFP; left) and ubiquitously-expressed myosin signal (Sqh::mCherry; right) in an egg chamber just prior to wave onset. **D**. Images from the same egg chamber following wave onset. **E**. Zoom-in on the cell highlighted by the arrow in **D**. Arrows in **D**,**E** point to waves. Scale bars: 50 μm (**A**-**C**) and 20 μm (**D**,**E**).

bioRxiv preprint doi: https://doi.org/10.1101/2023.11.06.565883; this version posted November 6, 2023. The copyright holder for this preprint (which was not certified by peer review) is the author/funder, who has granted bioRxiv a license to display the preprint in perpetuity. It is made available under aCC-BY 4.0 International license.

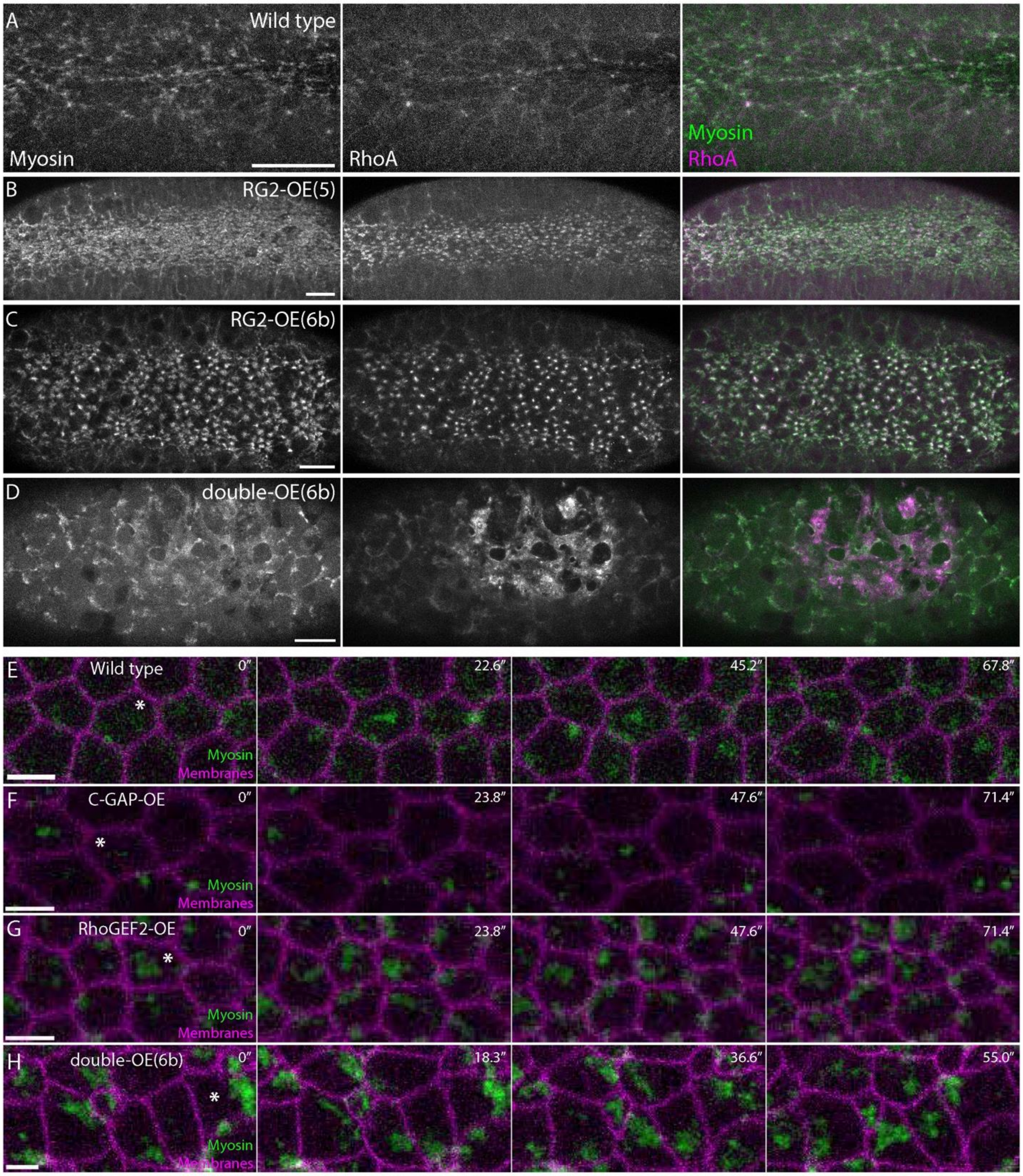

Supplemental Figure S3: RhoA and myosin change upon perturbation to RhoGEF2 and C-GAP levels.

A. MIPs of myosin (left), RhoA (center, visualized by the Rho-binding domain of anillin), and merged channels (right) in a wild-type embryo. Both proteins appear as spots within a supracellular network. **B**. Upon RhoGEF2 overexpression, both proteins form larger spots. **C.** Reorganization into spots is also seen in RhoGEF2-OE embryos in which gastrulation defects are

bioRxiv preprint doi: https://doi.org/10.1101/2023.11.06.565883; this version posted November 6, 2023. The copyright holder for this preprint (which was not certified by peer review) is the author/funder, who has granted bioRxiv a license to display the preprint in perpetuity. It is made available under aCC-BY 4.0 International license.

more severe. **D.** Upon overexpression of both regulators, myosin and RhoA reorganize into traveling waves rather than spots. The bright region in the RhoA signal is the residual cellularization front. **E**. Time series from a wild-type embryo showing myosin pulsing and becoming more prominent over time. **F**. Myosin pulses in C-GAP-OE embryos are smaller and build up less over time. **G**. Myosin pulses in RhoGEF2-OE embryos are larger but still accumulate over time. **H**. Myosin in double-OE embryos is organized in traveling waves instead of medioapical pulses. Asterisks in **E**-**H** highlight cells with prominent examples of pulses or waves. Images in **E**-**H** are surface projections of the myosin channel and a single optical section of the membrane channel. Scale bars: 20 µm (**A**-**D**); 5 µm (**E**-**H**).

bioRxiv preprint doi: https://doi.org/10.1101/2023.11.06.565883; this version posted November 6, 2023. The copyright holder for this preprint (which was not certified by peer review) is the author/funder, who has granted bioRxiv a license to display the preprint in perpetuity. It is made available under aCC-BY 4.0 International license.

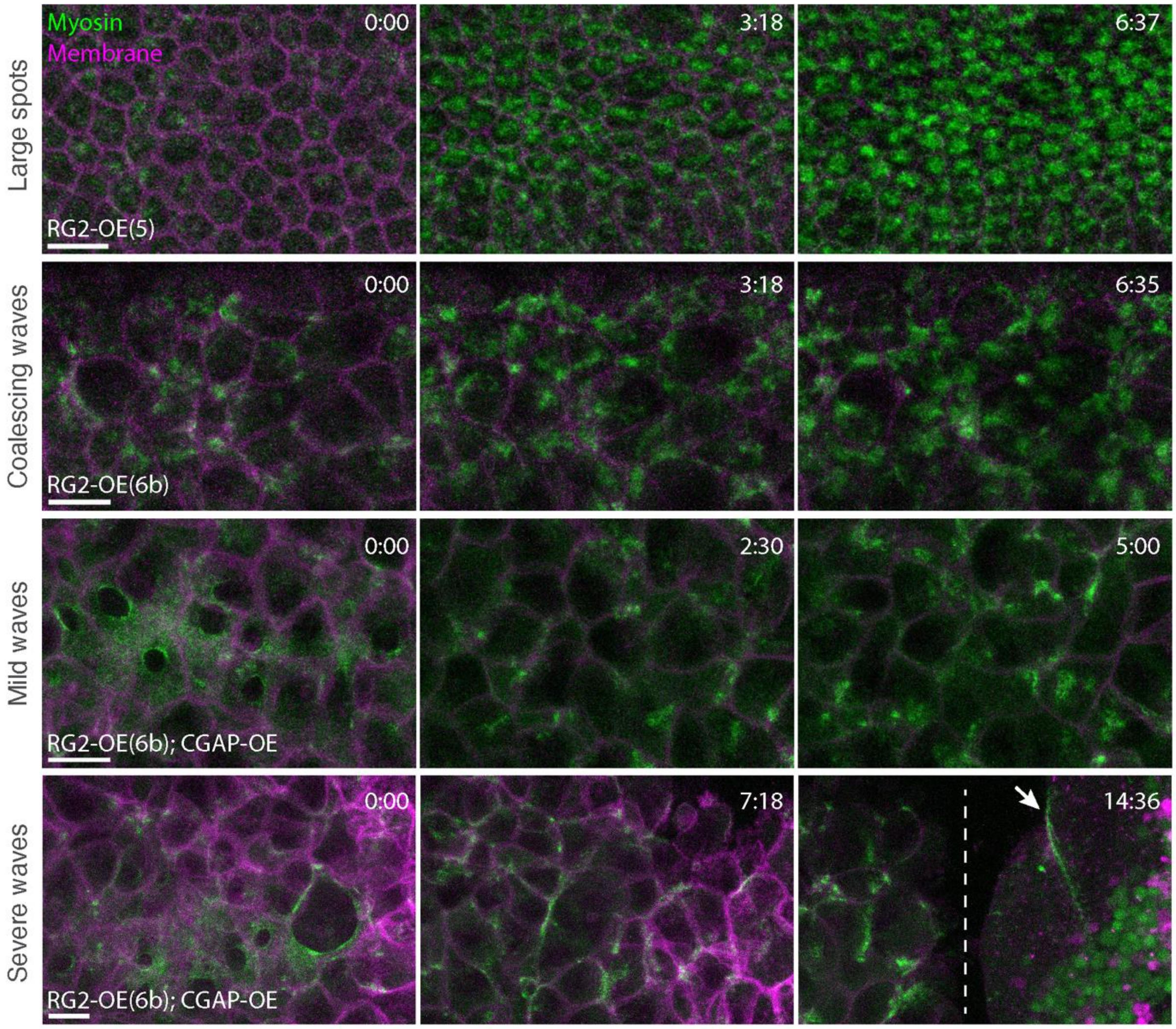

Supplemental Figure S4: Phenotype classes observed in RhoGEF2-OE and double-OE embryos.

MIPs of myosin (green) and membranes (magenta) at three time points for each of the four phenotype classes scored in Figure 5B,C. **A**. Accumulation of abnormally large medioapical spots ('large spots'). **B**. Waves that form early before transitioning into stable spots and/or a network ('coalescing waves'). **C.** Waves that appear in some cells after cellularization finishes in part of the embryo ('mild waves'). These cells are usually pulled towards one pole of the embryo due to cortical instability from cellularization failure at other locations. **D**. Waves that appear in embryos in which incipient cells break down before cellularization is complete in any part of the embryo ('severe waves'). Third panel shows a large-scale wave deforming the open cortex (arrow) next to the border between open cortex and partially-formed cells (dashed line). Scale bars: 10 µm. All timestamps are min:sec.

bioRxiv preprint doi: https://doi.org/10.1101/2023.11.06.565883; this version posted November 6, 2023. The copyright holder for this preprint (which was not certified by peer review) is the author/funder, who has granted bioRxiv a license to display the preprint in perpetuity. It is made available under aCC-BY 4.0 International license.

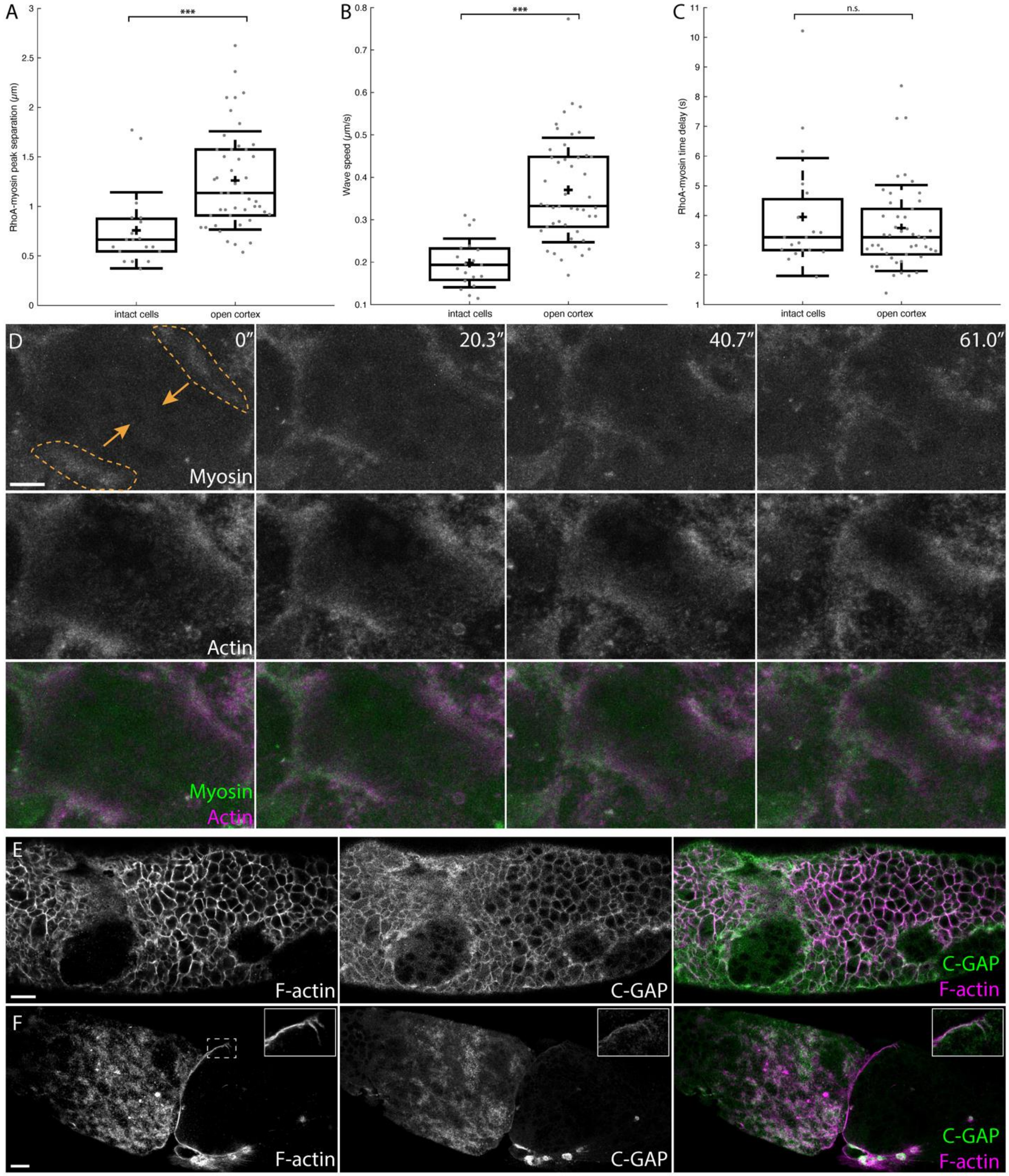

Supplemental Figure S5: Waves in intact cells and those in open cortex vary in speed but not RhoA-myosin delay, and C-GAP localization is similar to that of F-actin

A. Spatial separation between peaks of the RhoA and myosin wavefronts in intact cells and in the open cortex after cellularization fails. **B**. Wave speed for the same two classes of waves. **C**. RhoA-to-myosin delay time for the two classes of waves. All data for **A**-**C** are from double-

bioRxiv preprint doi: https://doi.org/10.1101/2023.11.06.565883; this version posted November 6, 2023. The copyright holder for this preprint (which was not certified by peer review) is the author/funder, who has granted bioRxiv a license to display the preprint in perpetuity. It is made available under aCC-BY 4.0 International license.

OE(6b) embryos; the same waves and embryos are used for all three plots. n = 19 waves from 4 embryos for intact cells and 46 waves from 6 embryos for open cortex. Using the Mann-Whitney U test and individual waves as samples, $p \sim 2.9 \times 10^{-5}$, 1.2×10^{-10}, and 0.72 for **A**, **B**, and **C**, respectively. Box plot components are as in Figure 3C, but without colored points for embryo means. **D.** MIPs of myosin (top), F-actin (middle), and merged channels (bottom) at four time points in a double-OE(6b) embryo. F-actin leads myosin slightly in the pair of converging waves outlined in orange. **E**. F-actin and C-GAP signals from a fixed embryo in which tears were just beginning to form in the epithelial layer. **F**. The same signals from an embryo in which the cells had almost entirely broken down, leaving an open cortex to the right of the image. Scale bars: 10 µm (**D**); 20 µm (**E**,**F**).

bioRxiv preprint doi: https://doi.org/10.1101/2023.11.06.565883; this version posted November 6, 2023. The copyright holder for this preprint (which was not certified by peer review) is the author/funder, who has granted bioRxiv a license to display the preprint in perpetuity. It is made available under aCC-BY 4.0 International license.

Video captions

Video S1: Effects of RhoGAP15B and Ect2 perturbation on actomyosin waves in nurse cells. Maximum-intensity projections of Sqh::GFP for egg chambers of the following six genotypes: wild-type, *RhoGAP15BΔRhoGAP/+*, RhoGAP15B RNAi, *pbl*^{3}*/+*, Ect2 RNAi, and Ect2-OE, corresponding to egg chambers used for Figure 2 and Supplemental Figure 1. All RNAi and overexpression constructs are germline-specific. Scale bars: 50 µm; time stamps are hr:min:sec; t=0 is used only for reference and is not standardized between movies. Time steps are different between movies and range from 12 to 30 seconds.

Video S2: Ect2 and RhoGAP15B exit the nucleus as dumping proceeds. First movie: MIP of Ect2 under control of the *sqh* promoter during dumping, showing exit from the nucleus in posterior nurse cells before anterior ones. Second movie: a similar pattern is seen with RhoGAP15B. Scale bars: 50 µm; time stamps are hr:min:sec. Time step is 1 minute for the first movie and 2.5 minutes for the second.

Video S3: Myosin contractions increase in intensity and become more wave-like as dumping proceeds. Two concatenated time series of the same egg chamber, showing progression in myosin contractility from local spots to waves that propagate across the cell. Scale bars: 50 µm; time stamps are hr:min:sec.

Video S4: Waves are observed in egg chambers expressing germline-specific Zip::GFP. MIPs from egg chambers expressing germline-specific Zip::GFP (left) and ubiquitous Sqh::GFP (right), showing waves are inside nurse cells, despite some overlying follicle-cell specific myosin dynamics. All movies are from the same egg chamber; scale bars: 50 µm in the first two movies and 20 µm in the last; timestamps are hr:min:sec. Time step in the last movie is 12 seconds and 20 seconds in the first two.

Video S5: Myosin behavior in mesoderm cells cells in the genotypes explored in this study. Myosin pulses appear in wild-type, C-GAP-OE, and RhoGEF2-OE embryos, although pulse size and degree of furrow formation vary with genotype. The two bottom double-OE embryos show relatively mild examples of the traveling wave phenotype, although cells begin to rip apart in the bottom-right movie. Scale bars: 20 µm; time stamps are min:sec. Time steps are different between movies and range from roughly 7 to 18 seconds.

Video S6: Large traveling waves that annihilate upon collision appear in the cortex remaining after cellularization failure in double-OE embryos. First movie: myosin signal in a double-OE(5) embryo, with remnant cells to the right. Second movie: zoom-in of myosin waves in a double-OE(6b) embryo, highlighting spiral wave patterns coexisting with planar waves, followed by a whole-embryo view of trains of planar waves. Third movie: myosin and RhoA in a double-OE(6b) embryo, from cellularization failure through emergence of large-scale planar waves, in which RhoA wavefronts can be seen to lead myosin wavefronts. Fourth movie: myosin and F-actin in a double-OE(6b) embryo, with remnant cells to the right. Myosin very slightly leads F-

bioRxiv preprint doi: https://doi.org/10.1101/2023.11.06.565883; this version posted November 6, 2023. The copyright holder for this preprint (which was not certified by peer review) is the author/funder, who has granted bioRxiv a license to display the preprint in perpetuity. It is made available under aCC-BY 4.0 International license.

actin in most waves as they gradually become organized into trains of wavefronts. Scale bars: 20 µm; time stamps are hr:min:sec. Time steps vary between movies and range from 9 to 25 seconds.
